# 1111. Case Study. Pediatric Hand Burns from Treadmill Friction: Small TBSA, High Morbidity, Preventable

**DOI:** 10.1093/jbcr/irag033.215

**Published:** 2026-04-06

**Authors:** Kyung Yoon, Amber Jin, Tamar Gordis, Kirill Antonov, Michael L Cooper

**Affiliations:** Northwell, Marietta, GA; Touro College of Osteopathic Medicine, Staten Island, NY; Northwell, Staten Island, NY; Northwell, Staten Island, NY; Staten Island University Hospital Northwell Health, Staten Island, NY

## Abstract

**Patient Presentation (age range, injury details, relevant history):**

Five pediatric patients (10 months–7 years 6 months; 3 males, 2 females) sustained treadmill friction burns at home while reaching for a ball or touching a moving treadmill belt. Burns involved dorsal and volar hands, digits, and wrists, ranging from partial- to full-thickness. TBSA was 1–3%. Functional impairment included pain-limited range of motion, with one patient showing early contracture. No patients had prior hand injuries or comorbidities.

**Clinical Challenges:**

Pediatric treadmill friction burns pose challenges despite small TBSA, often affecting multiple digits with high risk of pain-limited motion, stiffness, and contracture. Management decisions between surgery and conservative care were critical, with some requiring debridement and grafting. Long-term scar prevention included compression garments, silicone gels, intralesional steroids, and CO₂ laser. Pediatric-specific factors, such as compliance and caregiver education, compounded management. These preventable injuries highlight the need for early recognition and family counseling.

**Management Approach:**

Management of pediatric treadmill friction burns was based on burn depth, location, and functional impact. Superficial injuries were treated conservatively with topical therapy, while deep burns required debridement and split-thickness skin grafting. Multidisciplinary care included pain control, occupational therapy, and early ROM exercises. Scar management used compression garments, silicone gel, intralesional steroids, and, when needed, CO₂ laser for contractures. Follow-up ensured adherence to rehabilitation. This individualized approach allowed most patients to regain satisfactory hand function and minimize long-term morbidity.

**Outcomes:**

Despite small total body surface area, injuries caused significant functional morbidity. Most patients regained satisfactory hand function with individualized care. One developed flexion contractures of the palm, requiring CO₂ laser; others had mild, transient stiffness. Scar management—including compression garments, silicone gels, and intralesional steroids—minimized hypertrophic scarring. Multidisciplinary care combining acute management, surgery when needed, and rehabilitation facilitated recovery and limited long-term sequelae, highlighting the high morbidity of pediatric treadmill friction burns and the importance of early, coordinated management.

**Lessons Learned:**

Pediatric treadmill friction burns, though small in surface area, can cause deep injury and functional impairment. Early recognition and individualized management—including surgery when needed, scar prevention, and rehabilitation—are critical. Multidisciplinary care with caregivers and therapists improves recovery. These injuries are largely preventable, highlighting the importance of education, supervision, and home safety. Awareness of this mechanism guides clinical decisions and injury prevention.

**Applicability to Practice**:

